# Analysis of Radiology Report Recommendation Characteristics and Rate of Recommended Action Performance

**DOI:** 10.1001/jamanetworkopen.2022.22549

**Published:** 2022-07-22

**Authors:** Tiantian White, Mark D. Aronson, Scot B. Sternberg, Umber Shafiq, Seth J. Berkowitz, James Benneyan, Russell S. Phillips, Gordon D. Schiff

**Affiliations:** 1 Harvard Medical School, Boston, Massachusetts; 2 Department of Family Medicine, Oregon Health & Science University, Portland; 3 Department of Medicine, Beth Israel Deaconess Medical Center, Boston, Massachusetts; 4 Department of Radiology, Beth Israel Deaconess Medical Center, Boston, Massachusetts; 5 Healthcare Systems Engineering Institute, College of Engineering, Northeastern University, Boston, Massachusetts; 6 Harvard Medical School, Center for Primary Care, Boston, Massachusetts; 7 Center for Patient Safety Research and Practice, Brigham and Women’s Hospital, Boston, Massachusetts

## Abstract

**Importance:**

Following up on recommendations from radiologic findings is important for patient care, but frequently there are failures to carry out these recommendations. The lack of reliable systems to characterize and track completion of actionable radiology report recommendations poses an important patient safety challenge.

**Objectives:**

To characterize actionable radiology recommendations and, using this taxonomy, track and understand rates of loop closure for radiology recommendations in a primary care setting.

**Design, Setting, and Participants:**

Radiology reports in a primary care clinic at a large academic center were redesigned to include actionable recommendations in a separate dedicated field. Manual review of all reports generated from imaging tests ordered between January 1 and December 31, 2018, by primary care physicians that contained actionable recommendations was performed. For this quality improvement study, a taxonomy system that conceptualized recommendations was developed based on 3 domains: (1) what is recommended (eg, repeat a test or perform a different test, specialty referral), (2) specified time frame in which to perform the recommended action, and (3) contingency language qualifying the recommendation. Using this framework, a 2-stage process was used to review patients’ records to classify recommendations and determine loop closure rates and factors associated with failure to complete recommended actions. Data analysis was conducted from April to July 2021.

**Main Outcomes and Measures:**

Radiology recommendations, time frames, and contingencies. Rates of carrying out vs not closing the loop on these recommendations in the recommended time frame were assessed.

**Results:**

A total of 598 radiology reports were identified with structured recommendations: 462 for additional or future radiologic studies and 196 for nonradiologic actions (119 specialty referrals, 47 invasive procedures, and 43 other actions). The overall rate of completed actions (loop closure) within the recommended time frame was 87.4%, with 31 open loop cases rated by quality expert reviewers to pose substantial clinical risks. Factors associated with successful loop closure included (1) absence of accompanying contingency language, (2) shorter recommended time frames, and (3) evidence of direct radiologist communication with the ordering primary care physicians. A clinically significant lack of loop closure was found in approximately 5% of cases.

**Conclusions and Relevance:**

The findings of this study suggest that creating structured radiology reports featuring a dedicated recommendations field permits the development of taxonomy to classify such recommendations and determine whether they were carried out. The lack of loop closure suggests the need for more reliable systems.

## Key Points

**Question:**

What types of recommendations are contained in radiology reports, and how often are recommended follow-up actions carried out in the primary care setting?

**Findings:**

In this quality improvement study including 598 radiology reports at a single academic center, a taxonomy of recommended actions, time frames, and contingencies was developed. Application of this taxonomy showed that 1 of 7 recommendations were associated with failures to carry them out, and factors related to the report, patients, and clinicians were associated with successful vs unsuccessful follow-up.

**Meaning:**

The findings of this study suggest that no follow-up on action-requiring abnormalities represents an important patient safety and malpractice risk; structured reports may help codify recommended actions and identify lack of follow-up.

## Teaser

This quality improvement study examines the communication of findings between radiology and primary care and implementation of recommended follow-up actions.

## Introduction

A long-standing patient safety issue is the failure to close the loop on communication and implementation of actionable findings from radiologic tests.^1,2^ Follow-up recommendations are not uncommon in radiology reports. For example, recommendations for additional imaging (RAIs) are found in more than 10% of all reports, yet adherence rates range from 29% to 77%.^2,3,4^ Such lack of follow-up has the potential to cause substantial patient harm and malpractice risk, particularly by causing delayed diagnosis and treatment of cancers and other serious diagnoses.^3^

One factor in suboptimal follow-up is the lack of a standardized approach for highlighting actionable recommendations in radiology reports. Some have proposed placing a prioritized succinct list of relevant actionable recommendations within the summary or impression section of the report, given that many referring clinicians read only this section.^5,6^ Such placement, however, is not standard practice. In addition, when recommendations are contained in the summary or impression section, recommended actions can be obscured in long narratives summarizing findings and in the diagnostic conclusions.^6^ This variable placement of embedded information in the report poses challenges for referring primary care physicians (PCPs), who already face data overload.^7^

One step toward more reliable loop closure on actionable radiologic findings is developing an easier way to extract, code, and track recommendations.^8^ Three broad approaches have been attempted. Several health care organizations have piloted programs in which nonclinician staff manually query and track all actionable recommendations.^3,9^ Others have used natural language processing to harness recommendations from narrative radiology reports.^10,11,12,13^ A third approach involves structured reporting, whereby radiologists use a uniform format, a consistent organization, and standard terminology when entering recommendations.^14,15,16^ Even when more structured approaches were used, none applied a taxonomy that explicitly called for coded recommendations, time frame, and contingencies.^3,14,15,16,17,18,19^

Although recommendations is typically not a separate field in radiology reports, at the Beth Israel Deaconess Medical Center, the radiology department encourages radiologists to enter recommendations into a field created by the institution’s information technology department for actionable recommendations. As part of a larger project examining diagnostic loop closures,^20^ we were able to take advantage of this revised radiology report format to study these recommendations to create a new taxonomy of radiologist-recommended actions. We then used this new model to measure rates of loop closure both overall and within subcategories of our taxonomy to uncover features that are associated with more reliable loop closure rates.

## Methods

### Study Sample

In this quality improvement study, we examined radiology reports generated from studies performed on patients of a large primary care practice staffed by 65 primary care attendings, 109 residents, and 9 nurse practitioners from January 1 to December 31, 2018. Data analysis was conducted from April to July 2021. The study was approved by the institutional review board of Beth Israel Deaconess Medical Center and Harvard Medical School. We adhered to relevant portions of the Strengthening the Reporting of Observational Studies in Epidemiology (STROBE) reporting guideline for observational studies.

We identified 20 common imaging studies ordered at this clinic (eAppendix 1 in the Supplement). Mammography studies were excluded because abnormal mammography findings were tracked and followed up with a separate system. Only radiology reports with a radiologist’s recommendation (ie, recommendations field was not blank) were included. For loop closure analysis, we excluded recommendations with follow-up time frames longer than 25 months from the index study date.

### Taxonomy Development

All radiology recommendations were manually extracted from the recommendations field into an Excel (Microsoft Corp) worksheet qualitatively reviewed to identify common elements. Based on initial review of a sample of 100 cases, we deconstructed recommendations into 3 elements: (1) recommended action (eg, perform a different test, specialty referral), (2) time frame, and (3) any contingencies, defined as conditional clauses or qualifying statements (eg, if patient is a smoker, repeat computed tomography scan in 1 year).

### Review for Loop Closure

In reviewing for subsequent loop closure, we classified follow-up actions into 3 scenarios: (1) recommended action was performed; (2) there was documented disagreement by the referring physician with the recommended action, in which case we also classified the action as closed; and (3) the patient had died or there was documented patient refusal. We chose not to question the clinical judgment of either the radiologist or referring clinician, accepting at face value any physician documentation that a recommendation was deemed unnecessary as evidence of a closed loop. In all other cases, including when the patient cancelled or failed to keep scheduled appointments after a recommended follow-up had been scheduled, we considered the loop to be open.

Medical record review to determine whether loops were closed was conducted in 2 stages. First, the medical student researcher (T.W.) assessed loop closure using only structured data (a documented specialty clinic visit, radiology report for recommended follow-up test) without manually examining the free-text narrative progress notes, reports, or letters. This process was intended to streamline the study and limit the number of medical records needing more detailed manual review and providing a method that could be potentially automated in the future based on coded recommendations.

In the second stage, a detailed retrospective medical record review involving all of the electronic health record data was performed to evaluate all recommendations that remained open loops after the initial review (eAppendix 2 in the Supplement). Loop closure status was revised when appropriate (eg, if the PCP documented that the patient had recommended the test be performed elsewhere). In all cases in which loop closure was not clearly documented or determined, the case was rereviewed by 2 additional internists (M.D.A. and G.D.S.) who examined and adjudicated the cases.

### Statistical Analysis

Using R, version 4.0.2 (R Foundation for Statistical Computing), we performed analysis of factors associated with loop closure using 2-proportion *z* tests or Fisher exact tests to compare loop closure rates between closed vs open loop cases, with *P* = .05 as the cutoff level for determining statistical significance. For cases in which the loop was not closed within the recommended time frame, a quality improvement internist (M.D.A.) further reviewed the circumstances of the failure, assessed the clinical significance, determined whether patients needed additional follow-up at that point, and, if needed, referred the open loop to their PCP, and, where warranted, to the risk management quality improvement department.

## Results

Of 4911 eligible imaging studies, 532 reports (10.8%) generated by the radiology department for our primary care clinic contained a recommendation in the recommendations field. Reports lacking recommendations were not the focus of this study and thus were not reviewed. Among these 532 reports, the top imaging modalities were ultrasonography (248 [46.6%]), computed tomography (181 [34.0%]), and plain radiograph (89 [16.7%]) (Table 1). In 7 reports, the radiologist explicitly stated no further action was needed. Of the remaining reports, 461 (86.7%) had 1 recommended action, 58 (10.9%) had 2 recommended actions, and the remaining 6 (1.1%) had more than 2 recommended actions. In total, 598 actionable recommendations were extracted. We found 32 recommendations within sections other than the recommendations field. Within this group of embedded recommendations, 16 of the 32 (50%) had other recommended actions within the recommendations field. In another 10 recommendations (31%), the recommendations field simply referred the reader to a previous section of the radiology report.

**Table 1.** Type of Studies With Actionable Recommendations

Modality	Anatomic region	No. (%)
Computed tomography (n = 181)	Abdomen	10 (5.5)
	Abdomen and pelvis	61 (33.7)
	Chest	110 (60.8)
Magnetic resonance imaging (n = 14)	Abdomen	4 (28.6)
	Abdomen and pelvis	1 (7.1)
	Pelvis	4 (28.6)
	Renal	5 (33.3)
Ultrasonography (n = 248)	Abdomen	62 (25.0)
	Genitourinary	3 (1.2)
	Pelvis	84 (33.9)
	Renal	15 (6.0)
	Thyroid	84 (33.9)
Radiography (n = 89)	Chest	89 (100)

Using our taxonomy to classify recommendations (Table 2), we found that most involved RAIs in the form of an additional imaging study of a different modality or repeating the same study. Among non-RAIs, the most common was specialty referral, which was included as the recommended action in 119 (19.9%) of all recommendations. Examining recommended time frames for follow-up, 370 (61.9%) of the recommendations did not contain any specified time frame. Among recommendations with an explicit time frame, more than half contained time frames of 1 year or longer. A small number (28 [4.7%]) of recommendations had a time frame that was not explicit, for instance, repeat chest radiograph after antibiotic treatment is completed.

**Table 2.** Taxonomy of Recommendations (n = 598) vs Loop Closure Rates

Taxonomy concept	Recommendation details	No.	Loop closure %, initial^a^	Loop closure %, revised^b^
Recommended action				
RAI (n = 462)	Repeat same study	230	61.3	81.8
	Order additional radiology study	231	70.3	89.2
Non-RAI (n = 196)	Specialty referral	119	84.0	94.1
	Invasive image-guided procedure	47	85.1	95.7
	Evaluation for a specific clinical diagnosis	13	69.2	84.6
	Initiate treatment for a particular diagnosis	1	100	100
	Obtain prior imaging for comparison	9	44.4	100
Choice between 2 types of actions (n = 55)	Recommend, eg, pelvic ultrasonography or obstetrician/gynecologist referral	55	81.8	94.5
Time frame				
Yes (n = 228)	Explicit	200	57.1	79.6
	Due date <1 y from index date	91	74.7	87.4
	Due date ≥1 y from index date	125	49.3	76.1
	Implicit or vague	28	85.7	85.7
No (n = 370)	No time frame specified	370	74.3	91.6
Contingencies				
Yes (n = 147)	Does not require substantive clinical decision-making	79	44.3	82.3
	Requires more substantive clinical decision-making	31	54.8	77.4
	Nonspecific/vague contingency language (eg, consider or elective)	66	59.1	83.3
No (n = 451)	No contingency language	429	76.5	89.7

Abbreviation: RAI, recommendations for additional imaging.

^a^ Based on test or appointment data in electronic medical record.

^b^ Revised after full medical record review.

Of the 598 recommendations, 147 recommendations (24.6%) contained contingencies. Contingency language was broadly classified into 3 categories: (1) advised exercise of clinical judgment and/or with further direct communication or consultation between the radiologist and the PCP to determine the necessity of the recommended action, (2) invoked decision-making criteria for information that can be relatively easily determined using information from the electronic health record or the patient without requiring substantial clinical judgment by a physician (eg, repeat computed tomography scan if the patient smokes), and (3) vague qualifying statements, such as could consider or elective (eTable in the Supplement).

After applying exclusion criteria, 593 recommendations were included in our loop closure analysis. Based on review of structured electronic data (our first-pass review) of recommended tests and referrals, we identified 410 recommendations (69.1%) as clearly being closed. Another 108 loop closures were found only on detailed medical records reviews, bringing the overall loop closure rate to 87.4%. Although reviewing structured test and appointment data alone was sufficient in tracking loop closure in 85.3% of closed loops for unconditional recommendations, only 63.2% of closed loops involving a contingency were trackable with this form of review.

Several factors were associated with loop closure rates. One was evidence of direct communication between the radiologist and referring PCP (eg, telephone or email messaging). If such communication was documented, the loop closure rate was 93.6% compared with 84.8% (*P* = .002) if no communication was noted.

We found 32 recommendations embedded in the larger report. For these recommendations, loop closure rate was significantly lower (63.6% vs 88.6%; *P* = .02) when the recommendations were part of the dedicated recommendations field. Elements of the taxonomy that were associated with varying rates of loop closure included length of the time frame, presence of contingency language, and type of action. When the specified time frame for completing follow-up was longer than 1 year, for instance, fewer loops were closed (76.1% vs 87.4%; *P* = .02). Compared with unconditional recommendations, recommendations with a contingency were closed at a lower rate (81.9% vs 89.7%; *P* = .004). In addition, recommendations in which the action involved RAIs were completed at a lower rate compared with ones involving non-RAIs (85.2% vs 92.9%; *P* = .02).

To better understand the nature of closed and open loops, we further classified them into subcategories (Table 3). One aspect of potential interest is timing related to the recommendation and loop closure. We allowed a flexible time window to permit patients to follow through with the recommendations, allowing a time frame one-third longer or shorter than the radiologist-recommended time frame (eg, if 6-12 months was the specified range, any loop closure between 4 and 16 months from the index study date was considered as on time). With this classification, 75% of loop closures occurred within the specified time frame and 25% occurred before or after the specified time. In several cases in which the loop was closed earlier than expected, medical records review of these earlier closures suggested that the loop may have been incidentally rather than deliberately closed to adhere to the recommendations. For example, 1 index chest radiograph revealed enlarged hilar lymph nodes. The radiologist recommended monitoring by repeating the study in 4 to 6 weeks to rule out cancer, but a second radiograph and additional chest computed tomography scan were performed 1 week later when the patient presented acutely to the emergency department for chest pain and fever. However, among recommendations with no specified time frame, most (ie, 67.6%) of the loops were closed by 30 days from the index study date and, by 3 months, 88.2% were closed.

**Table 3.** Subtypes of Closed and Open Loops

Loop status	Scenario	Conditional, No.	Unconditional, No.	Total, No.
**Loop closed**				
Structured data available demonstrating completion of recommended action	Explicit time frame specified: action completed within specified time	9	75	84
	Explicit time frame specified: action completed before specified time	7	13	20
	Explicit time frame specified: action completed after specified time	2	6	8
	No explicit time frame: action completed within 25 mo of index study	73	225	298
No structured data available; medical record review demonstrated completion of recommended action	Clinician judged recommended action was not warranted given clinical context	40	5	45
	Contingency specification met (eg, patient is not a smoker)	25	NA	NA
	Alternative follow-up action taken	5	23	28
	Recommended action completed but not captured during initial review of structured data	5	22	27
	No follow-up recommended by radiology on manual review	1	3	4
	Patient died or refused recommended action	2	2	4
No.		53	55	108
**Loop not closed** ^a^				
No structured data available; medical record review demonstrated failure to complete recommended action	No consensus plan made for follow-up of radiologic findings	18	8	26
	Consensus plan developed but no follow-up ordered and/or scheduled	12	31	43
	Patient did not present or cancelled scheduled follow-up appointment or test	2	4	6
No.		32	43	75

Abbreviation: NA, not applicable.

^a^ No evidence of recommended action being done.

Among loop closures that required review of unstructured free-text data, the 2 largest categories, making up 67.6% of all cases, were that the referring clinician felt that the recommended follow-up was unnecessary in the clinical context and the referring clinician took an alternative action (eg, referred a patient to the endocrine thyroid nodule clinic instead of the recommended annual ultrasonography surveillance).

Based on our review of these 598 cases and the literature,^4,5,9,10,11,12,13,14,15,16,17^ we created a framework outlining steps required for loop closure and mapped the various failure modes we observed to this model (Figure). In an ideal world, once a recommendation is made by radiology, this information would be received by the ordering PCP who would then interpret the findings in the appropriate clinical context, sometimes in consultation with the radiologist to decide how to best proceed. Next, on joint decision-making with the patient, a consensus would be reached on the optimal desired follow-up plan. This plan would then be executed and the loop completely closed when information from that follow-up (eg, a biopsy ruling out cancer) is fed back to the tracking system, which could be a radiologist, population manager, or PCP. This final step is beyond the scope of our present study but would be of interest for future studies to explore given that such feedback has the potential to help individual radiologists track and improve their performance.^6,21^

**Figure.**
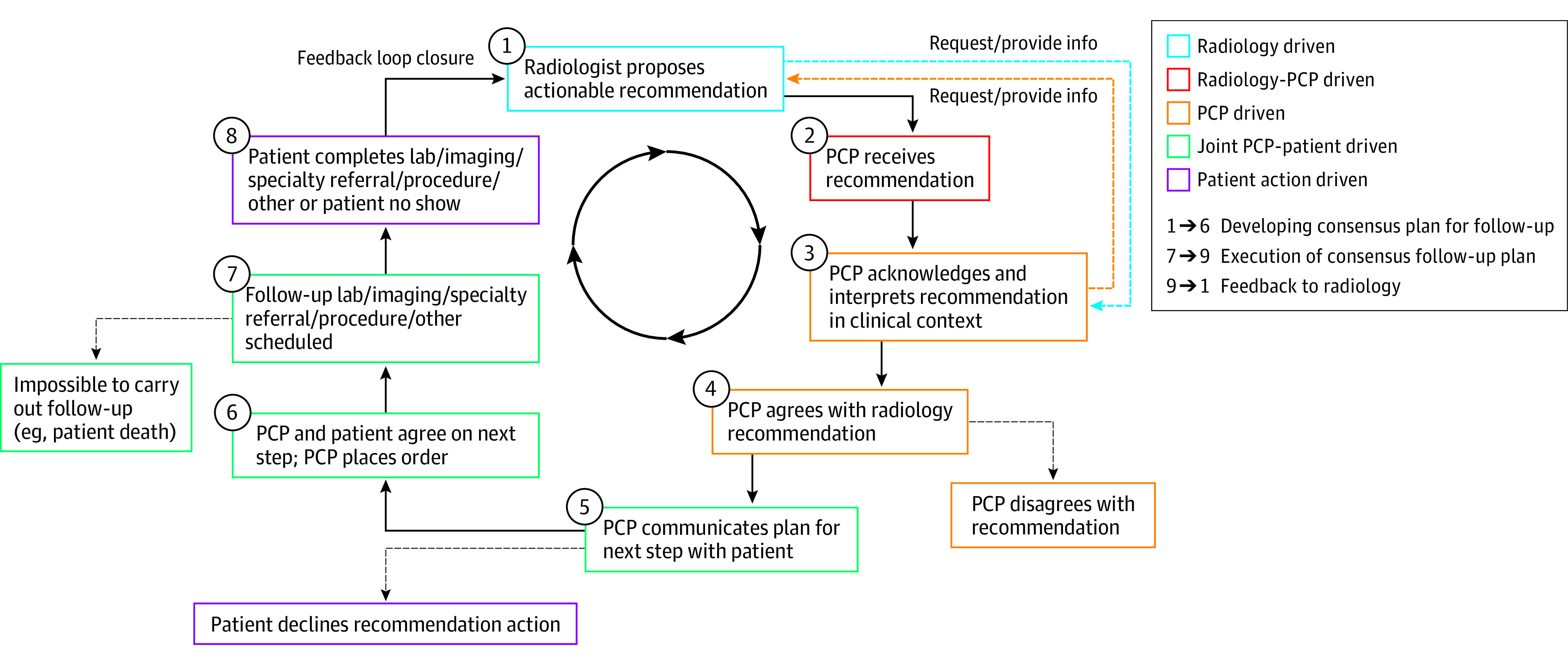
Conceptual Steps in Loop Closure on an Actionable Radiology Recommendation Steps 1 to 6: developing consensus plan for follow-up, steps 7 to 9: execution of follow-up plan, and steps 9 to 1: feedback to radiology. PCP indicates primary care physician.

Analyzing the 75 open loops in our sample, we found that loop closure was unsuccessful most frequently during the execution (carrying out the recommendation action) stage. Thus, in 57.3% of the open loops, there was documented agreement among all parties involved to proceed with a follow-up plan, but ordering and/or scheduling the needed action was not performed (Table 3). Only 8% of open loops arose from documented patient cancellation or no-show. The fraction of clinically worrisome open loop cases that posed a clinically significant patient safety risk was small (31 [5.2%]). Typically, such worrisome loops were for lesions suggestive of new cancers with no evidence of follow-up.

In 28% of all open loops, it appears that usual interventions fell far short in reliably closing these loops. These occasions included cases in which (1) repeated attempts by clinician teams to remind the patient to schedule a follow-up appointment (n = 7) were unsuccessful, (2) the patient appeared to have left the Beth Israel Deaconess Medical Center system soon after the index study (n = 10), and (3) completing follow-up was challenging owing to complicating medical illnesses (n = 4). One identifiable factor from medical records reviews that may have contributed to unsuccessful loop closure was transition of care (n = 9) when there were discontinuities in patient hand-off either due to a PCP leaving the practice or the physician who initially saw the patient and ordered the incident radiology study was not the patient’s regular PCP.

## Discussion

Although literature has classified radiology recommendations according to some of the domains in our taxonomy,^3,10,14,15,16,17,18^ this report is, to our knowledge, the first on the development and application of a comprehensive tripartite taxonomy, including contingencies and subcategories that help quantify each subcategory in relation to loop closure. Such a taxonomy can be helpful in developing a coded system that could facilitate automatic tracking for closing the loops on different types of recommendations. Our findings suggest that, if the system is properly designed, as many as 80% of loop closures could be electronically documented, coded, and automatically tracked using structured electronic health record data without having to resort to manual review of medical records.

The overall loop closure rate in our sample of 87.4% was considerably higher than rates reported in the literature.^3,4,10,11,16,17,18,19,22,23^ This difference may stem from our meticulous efforts to conduct detailed medical records reviews to detect other details or factors to label a recommendation loop as closed. More importantly, this higher rate may reflect the radiology department’s efforts to place actionable recommendations into a separate section of the report. While it is beyond the scope of this study to directly compare loop closure rates between our sample and the rates in a study in which reports lack such a dedicated section, our finding that recommendations that were embedded in a different section had a lower loop closure rate is suggestive. Another potential contributor may be that all patients in our sample by virtue of our inclusion criteria had an established PCP—a factor that has been shown to correlate with higher follow-up adherence for incidental radiologic findings.^2^

Nonetheless, 1 in 7 patients with major radiologic abnormalities for whom radiology recommended follow-up actions did not have documented closure of these recommended steps. Although not all these lapses in follow-up were judged to be clinically significant, the rate is substantial and concerning given the large volume of radiology studies, many with important or incidental findings requiring follow-up.

Our study suggests that unsuccessful loop closures are often multifactorial, which is not surprising considering the number of steps and parties involved to close the loop. Although the bulk of published work on improving loop closure in radiology has focused on the neglect to pass information in a timely fashion from the radiologist to referring physician,^2,24^ our findings suggest additional failure modes and areas for improvement, such as including clearer, perhaps less conditional recommendations.^23^ Given data overload faced by busy PCPs, relying solely on their manual efforts to track, order, and ensure scheduling of a follow-up test (especially when follow-up is due far in the future) is suboptimal. Although the relatively high loop closure rate reported herein is a testament to the efforts by PCPs, future work applying a system engineering approach is needed to better understand workload and vulnerable steps in our current systems.^3,11,20^

Our findings suggest that improving the specificity of recommendations may help reduce failures in loop closure, particularly with recommendations containing contingencies. Like past published work, we found contingency language, often vaguely worded, to be associated with lower rates of loop closure.^17,25^ When faced with such conditional language, more failures occurred in developing a follow-up plan. More explicit recommendations minimizing the use of vague contingencies, such as "if warranted depending on clinical correlation" as suggested by others,^6,25,26^ would also facilitate tracking because both existing manual and natural language processing tracking approaches tend to exclude conditional recommendations.^3,10,27,28^

### Limitations

This study has limitations. First, it was based at a single institution with a unique electronic health record system and included only tests ordered by PCPs. Second, the manual reviews were performed by 1 reviewer, although all questionable cases were rereviewed by 1 of 2 experienced quality and safety clinicians. Third, as with all retrospective medical records reviews, determination of loop closure relied on documented evidence in the electronic health record. Hence, it is possible that some loops were misclassified because of missing documentation. Fourth, this study only examined closure of the recommendation loop but not necessarily of the clinical loop. For example, if a thyroid nodule was found on ultrasonography and radiology recommended referral to the endocrine clinic, we deemed that loop closed when the patient kept the clinic appointment regardless of whether or when the nodule was conclusively diagnosed as showing a malignant or benign lesion.

## Conclusions

Using a dedicated recommendation text field and a taxonomy to classify and track diagnostic loop closures, this study noted that follow-up was not performed for 1 in 7 recommended actions. Although multiple factors appeared to be associated with such unsuccessful follow-up, our findings suggest several potential areas for improvement, including dedicated radiology report recommendations fields; clearer, perhaps less conditional recommendations; better systems for scheduling patients for recommended follow-up examinations and referrals; more reliable systems for patient hand-off for PCPs who leave practices; and follow-up of patients who do not schedule or present for scheduled activities.

## Notes

Supplement. **eAppendix 1.** List of Imaging Studies **eAppendix 2.** Medical Record Review Instrument **eTable.** Full List of Contingencies Found
