# Supplementary material for: Analysis of Radiology Report Recommendation Characteristics and Rate of Recommended Action Performance
Source: JAMA Netw Open. 2022 Jul 22;5(7):e2222549. doi: 10.1001/jamanetworkopen.2022.22549 (PMC9308057; doi:10.1001/jamanetworkopen.2022.22549)
Supplement: Supplement. — eAppendix 1. List of Imaging Studies eAppendix 2. Medical Record Review Instrument eTable. Full List of Contingencies Found [file jamanetwopen-e2222549-s001.pdf]

Supplementary Online Content

White T, Aronson MD, Sternberg SB, et al. Analysis of radiology report recommendation characteristics and rate of recommended action performance. *JAMA Netw Open*. 2022;5(7):e2222549. doi:10.1001/jamanetworkopen.2022.22549

eAppendix 1. List of Imaging Studies

eAppendix 2. Medical Record Review Instrument

eTable. Full List of Contingencies Found

This supplementary material has been provided by the authors to give readers additional information about their work.

© 2022 White T et al. *JAMA Network Open.*

eAppendix 1. List of imaging studies

1. CT ABDOMEN W/C
2. CT ABD W&W/O CONTRAST
3. CT ABD & PELVIS WITH CONTRAST
4. CT ABD & PELVIS W & W/O CONTRAST, ADDL SECTIONS
5. CT ABD & PELVIS W/O CONTRAST
6. CT CHEST W/O CONTRAST
7. CT CHEST W/CONTRAST
8. CHEST (PA & LAT)
9. CHEST (SINGLE VIEW)
10. THYROID U.S.
11. PELVIS, NON-OBSTETRIC
12. RENAL U.S.
13. ABDOMEN US (COMPLETE STUDY)
14. COMPLETE GU U.S. (BLADDER & RENAL)
15. MRI ABDOMEN W/O CONTRAST
16. MR ABDOMEN W/CONTRAST
17. MR PELVIS W&W/O CONTRAST
18. MRI (ABDOMEN & PELVIS) W&W/O CONTRAST
19. MRI RENAL W&W/O CONTRAST
20. MRI ABDOMEN W/O & W/CONTRAST

© 2022 White T et al. *JAMA Network Open.*

eAppendix 2. Medical Record Review Instrument

<u>Confirm accuracy of open loop</u>

Is it truly open?

Errors in the download data?

Failure to capture "outside" or other information

Other

<u>Record basic information</u>

Sentinel test case being reviewed here

Sentinel date - specified due timeframe for recommended action

Sentinel abnormal result

<u>Assessment of Details and Reason(s) for Open Loop</u> (to extent possible from chart)

Clinical context / issue

EX.

Past hx of elevated malignancy

Hospice patient, etc

Why was test, referral being done

Failure modes

Where is the failure mode: (at what step in the process did failure occur)?

EX. Appointment never booked; booked but "no show"?

Why did follow-up breakdown (any discernible, or even speculated reasons evident)?

<u>Outcomes</u>

Any adverse impacts (ED visit, worsening, late-stage cancer)

© 2022 White T et al. *JAMA Network Open.*

eTable. Full list of contingencies found

Contingencies	Classification
2nd repeat at 18-24mo optional if low risk and required if high risk pulmonary nodule per Fleischner guidelines	Does not require substantive clinical decision-making
Correlation with patient's smoking history, as clinically indicated	Does not require substantive clinical decision-making
if biopsy is not performed	Does not require substantive clinical decision-making
if colonoscopy not previously performed	Does not require substantive clinical decision-making
if cyst with internal flow persists	Does not require substantive clinical decision-making
if echogenic component persists within complex right ovarian cyst in 1 wk	Does not require substantive clinical decision-making
if high risk pulmonary nodule per Fleischner guidelines	Does not require substantive clinical decision-making
if high-risk patient (high-risk not defined)	Does not require substantive clinical decision-making
if no prior available for comparison and not previously biopsies	Does not require substantive clinical decision-making
if not recently performed	Does not require substantive clinical decision-making
if patient continue to feel the lump	Does not require substantive clinical decision-making

© 2022 White T et al. *JAMA Network Open.*

if patient is a smoker	Does not require substantive clinical decision-making
if patient is postmenopausal	Does not require substantive clinical decision-making
if patient is symptomatic for further evaluation	Does not require substantive clinical decision-making
if pt can tolerate	Does not require substantive clinical decision-making
If surgical management for the enlarging complex native right renal cyst is not considered	Does not require substantive clinical decision-making
if symptoms persist	Does not require substantive clinical decision-making
If the lesion is not surgically removed	Does not require substantive clinical decision-making
if the patient is 55-77 years old, has a smoking history of greater than 30 pack-years and has smoked within the past 15 years	Does not require substantive clinical decision-making
If the patient is 55-77 years old, has a smoking history of greater than 30 pack-years and has smoked within the past 15 years	Does not require substantive clinical decision-making
if there are referable pulmonary signs or symptoms	Does not require substantive clinical decision-making
if tolerated	Does not require substantive clinical decision-making
in high risk patient	Does not require substantive clinical decision-making
in high risk pt	Does not require substantive clinical decision-making

© 2022 White T et al. *JAMA Network Open.*

presuming the patient's clinical condition does not worsen	Does not require substantive clinical decision-making
Unless this was a clearly larger lesion recently and is resolving	Does not require substantive clinical decision-making
upon resolution of acute symptoms	Does not require substantive clinical decision-making
can be considered	“Consider” or “Elective”
can be performed	“Consider” or “Elective”
consider	“Consider” or “Elective”
could be considered	“Consider” or “Elective”
could be further assessed	“Consider” or “Elective”
could consider	“Consider” or “Elective”
elective	“Consider” or “Elective”
may be considered	“Consider” or “Elective”
may be obtained	“Consider” or “Elective”
maybe helpful	“Consider” or “Elective”
might be considered	“Consider” or “Elective”
optional	“Consider” or “Elective”
possible	“Consider” or “Elective”

© 2022 White T et al. *JAMA Network Open.*

can be done	“Consider” or “Elective”
could be further evaluated	“Consider” or “Elective”
as clinically indicated	Requires substantive clinical decision-making
clinical correlation	Requires substantive clinical decision-making
consider as clinically indicated	Requires substantive clinical decision-making
consider if clinical concern of ureteral lesion persists	Requires substantive clinical decision-making
could be of additional diagnostic value in evaluation of periurethral diverticulum	Requires substantive clinical decision-making
depending upon clinical circumstances, if sampling required	Requires substantive clinical decision-making
if applicable	Requires substantive clinical decision-making
if clinical concern persists	Requires substantive clinical decision-making
if clinically appropriate	Requires substantive clinical decision-making
if clinically indicated	Requires substantive clinical decision-making
if clinically warranted	Requires substantive clinical decision-making
if concern for small lung nodules	Requires substantive clinical decision-making
if continued clinical concern	Requires substantive clinical decision-making
if continued concern for a solid renal mass	Requires substantive clinical decision-making

© 2022 White T et al. *JAMA Network Open.*

if cysto-vaginal fistula is the clinical concern	Requires substantive clinical decision-making
if desired for further characterization of the right adnexal structure	Requires substantive clinical decision-making
if further evaluation is warranted	Requires substantive clinical decision-making
if ongoing concern for IBD	Requires substantive clinical decision-making
If the findings do not correlate with clinical history	Requires substantive clinical decision-making
if there are any clinical findings to suggest malignancy	Requires substantive clinical decision-making
If there is any concern for a GYN malignancy based on symptoms; if clinically relevant	Requires substantive clinical decision-making
If there is clinical suspicion for the possibility of a small pulmonary nodule	Requires substantive clinical decision-making
if there is continued clinical concern	Requires substantive clinical decision-making
If there is strong suspicion of pneumonia and additional radiographic confirmation is needed	Requires substantive clinical decision-making

© 2022 White T et al. *JAMA Network Open.*
